# A Hybrid Approach for Sports Activity Recognition Using Key Body Descriptors and Hybrid Deep Learning Classifier

**DOI:** 10.3390/s25020441

**Published:** 2025-01-13

**Authors:** Muhammad Tayyab, Sulaiman Abdullah Alateyah, Mohammed Alnusayri, Mohammed Alatiyyah, Dina Abdulaziz AlHammadi, Ahmad Jalal, Hui Liu

**Affiliations:** 1 Department of Computer Science, Air University, Islamabad 44000, Pakistan; 230245@students.au.edu.pk; 2 Department of Computer Engineering, College of Computer, Qassim University, Buraydah 52571, Saudi Arabia; salateyah@qu.edu.sa; 3 Department of Computer Science, College of Computer and Information Sciences, Jouf University, Sakaka 72388, Saudi Arabia; maalnusayri@ju.edu.sa; 4 Department of Computer Science, College of Computer Engineering and Sciences, Prince Sattam Bin Abdulaziz University, Al-Kharj 16278, Saudi Arabia; m.alatiyyah@psau.edu.sa; 5 Department of Information Systems, College of Computer and Information Sciences, Princess Nourah bint Abdulrahman University, P.O. Box 84428, Riyadh 11671, Saudi Arabia; daalhammadi@pnu.edu.sa; 6 Department of Computer Science and Engineering, College of Informatics, Korea University, Seoul 02841, Republic of Korea; 7 Cognitive Systems Lab, University of Bremen, 28359 Bremen, Germany

**Keywords:** machine learning, silhouettes, extremal regions, joint points, scalable key points

## Abstract

This paper presents an approach for event recognition in sequential images using human body part features and their surrounding context. Key body points were approximated to track and monitor their presence in complex scenarios. Various feature descriptors, including MSER (Maximally Stable Extremal Regions), SURF (Speeded-Up Robust Features), distance transform, and DOF (Degrees of Freedom), were applied to skeleton points, while BRIEF (Binary Robust Independent Elementary Features), HOG (Histogram of Oriented Gradients), FAST (Features from Accelerated Segment Test), and Optical Flow were used on silhouettes or full-body points to capture both geometric and motion-based features. Feature fusion was employed to enhance the discriminative power of the extracted data and the physical parameters calculated by different feature extraction techniques. The system utilized a hybrid CNN (Convolutional Neural Network) + RNN (Recurrent Neural Network) classifier for event recognition, with Grey Wolf Optimization (GWO) for feature selection. Experimental results showed significant accuracy, achieving 98.5% on the UCF-101 dataset and 99.2% on the YouTube dataset. Compared to state-of-the-art methods, our approach achieved better performance in event recognition.

## 1. Introduction

The field of computer vision is in a way dependent on the recognition of events, which involves observing, identifying, and locating the spatiotemporal structure in sequential events recorded from unstructured data sources like images as well as videos [1,2,3,4]. In this case, this is complex because it has several factors that affect its performance, such as lighting conditions, body posture, and variations in context, as well as camera perspectives [5,6,7]. The complexity is increased by video sequences which are full of multiple-object interactions and continuous movements [8,9]. Foundational to event recognition is the ability to identify key artifacts that are relevant to specific scenarios and track them throughout the video data [10,11]. This has wide-ranging applications, from security surveillance and sports training to smart indoor environments, reflecting how important it is for modern-era technological advancements [3,12].

Despite the advancements made in event recognition, there are still some challenges and limitations [13,14,15], especially when it comes to pose estimation and event recognition itself. Variations in human movement dynamics and diversity in body postures may hinder accurate pose estimation [16,17]. This study addresses the challenge of improving event recognition accuracy by incorporating advanced feature extraction and classification methods. We propose a framework that estimates a set of key body points for tracking and monitoring their presence within complex events. To achieve this critical body part detection for event identification, we employ a combination of feature descriptors. An amalgamation of advanced techniques is employed for feature extraction, including MSER, SURF, BRIEF, FAST, Optical Flow, DOF, distance transform, and HOG. These methods collectively capture both [18,19] spatial and temporal features essential for accurate event recognition. In the classification stage, a hybrid classifier is utilized due to its robustness and capability to manage high-dimensional data. Furthermore, a Grey Wolf Optimizer (GWO) is integrated to fine-tune the model parameters by enhancing the recognition system’s overall performance and adaptability.

Section 2 offers a thorough literature review that sets the stage for our research. In Section 3, we discuss the architecture of the proposed model, highlighting its distinctive features. Section 4 covers the experiments we carried out, along with a detailed analysis of the results. Section 5 explains some of the limitations of our research group. Finally, Section 6 concludes the study with key insights and recommendations for future research. This layout ensures a smooth and logical flow throughout the paper.

## 2. Literature Review

Detecting objects within images is an important step for event recognition in computer vision tasks. Several feature descriptors can be used to identify body parts in images to help detect events such as SURF (Speeded-Up Robust Features) [20], BRIEF (Binary Robust Independent Elementary Features) [21], MSER (Maximally Stable External Regions) [22], HOG (Histogram of Orientated Gradients) [23], DOF (degree of freedom), distance transform, FAST (Features from Accelerated Segment Test), and Optical Flow. Each of these descriptors uses different techniques for feature extraction such as defining interest points in SURF, computing the binary strings of image patches in BRIEF, identifying the extremal regions in images in MSER, and calculating the number of gradient orientations within a portion of an image, etc. Combining multiple descriptors will enhance the ability to extract features, as descriptors follow different approaches to extract features.

Event recognition relies on several classification algorithms to categorize events within data [24]. There is no single best algorithm for all data types. Classification algorithms use predefined rules to identify specific events, which leads to easier implementation [25]. In addition, the used algorithms are divided into different groups based on their methods including pattern matching, machine learning (ML), and deep learning. Every algorithm has special features that make it the best in dealing with specific types of data. The following is an overview of the popular classification algorithms used in event recognition, including Random Forest, support vector machines (SVMs), and Convolutional Neural Networks (CNNs).

Random Forest has been used in various applications due to its capability of handling non-linear tasks efficiently [26]. As a method based on ML, it has the ability to handle large datasets with advantages such as ease of learning, fast prediction, and resilience in dealing with imbalanced datasets. Moreover, the support vector machine (SVM) is one of the most powerful classification algorithms, which aims to make boundaries for decisions between two classes by enabling the estimate from at least one feature vector. SVMs have recently been used widely in research related to recognition [27]. According to [28], SVMs are powerful in dealing with complex datasets such as face detection and credit card fraud. Finally, yet importantly, a neural network (NN) is a network of interconnected processing units, inspired by the animal neuron [29,30]. Convolutional Neural Networks (CNNs) are analogs to Artificial Neural Networks [31]. CNNs have reported achieving brilliant results, which makes them one of the best methods in the deep learning field [32,33,34,35]. Thanks to advancements in computer vision powered by Convolutional Neural Networks, the things that can be achieved now would have seemed like science fiction in the past, like identifying faces in photos, robotics [36,37,38,39], automatic surveillance systems [40,41,42], developing self-driving cars, automating checkout at stores [43,44,45], and even creating smarter medical tools. However, understanding complex image content is key to event recognition, and CNNs have the capability to handle it effectively.

YOLOv8 is an extension of the YOLO framework, which is built for object detection in real time. The YOLOv8 includes several models, each of which can be used for different tasks: YOLOv8 for detection, YOLOv8-seg for segmentation, YOLOv8-pose for pose/key points detection, YOLOv8-obb for oriented detection [46,47], and YOLOv8-cls for classification [48]. While YOLOv8 provides excellent real-time performance in accurately detecting objects, the series of YOLO still faces several challenges such as handling occluded objects, detecting small objects, and adapting to dynamic environments. YOLOv8, in particular, limits the training model to using only images with resolutions below 1280 pixels and does not provide Post-Training Quantization, which can be a limitation when resource efficiency is critical.

In more recent advancements, deep learning has significantly evolved in event and human action recognition tasks [49,50,51,52]. Shrestha and Pandey [53], in their work titled “Human Action Recognition using Deep Learning Methods”, demonstrated how state-of-the-art approaches, particularly with deep learning models such as 3D CNNs and LSTM-based architectures, achieved higher accuracy in human action recognition compared to conventional techniques. Their study emphasizes the ability of modern CNN architectures, especially those augmented with temporal data, to capture both spatial and temporal features, thus improving the overall performance in event detection tasks. This trend highlights the shift towards more [54,55,56] robust and high-performing models in the field of event recognition, as these models increasingly outperform traditional methods in handling complex data scenarios involving motion and real-time action recognition. Integrating these advancements into the existing CNN-based approaches will ensure a more comprehensive understanding of the recent state-of-the-art techniques in event detection and classification.

## 3. Materials and Methods

**Figure 1:**
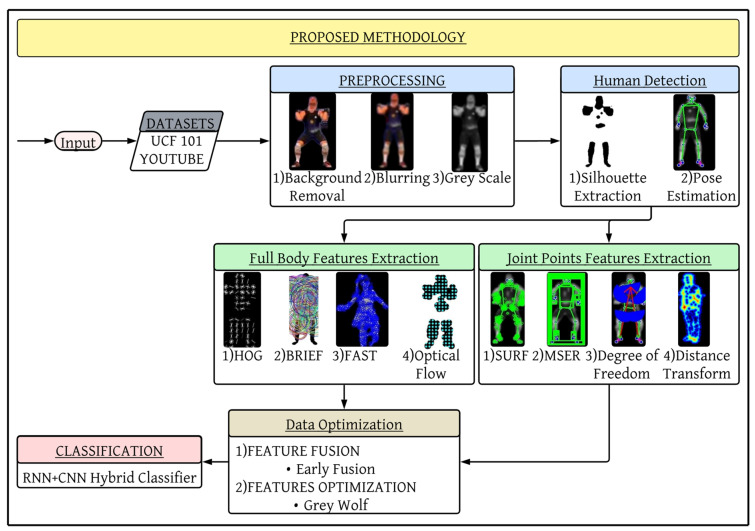
Proposed architecture for event classification.

Here, we describe the steps that we apply to study and forecast a person’s position, his/her motion, gait, and range of motion to judge further behavior. Our proposed systematic methodology starts by extracting the frames from a video. In the pre-processing stage, methods like the Gaussian blur filter and grayscale [57,58,59,60] image conversion are applied to remove noise and distracting backgrounds employing the rembg algorithm. This is achieved by silhouette extraction as well as human pose estimation. Next, we apply BRIEF, Optical Flow, FAST, and HOG to the significant points derived through the use of the application of MSER, SURF, distance transform, and DOF on their silhouettes and the whole human body, respectively. All these feature point values are merged using early fusion, and to dimensionally reduce the data, a Grey Wolf Optimizer is used. Last but not least, we use the CNN + RNN Hybrid Classifier for classification, which allows us to diagnose patterns and better predict the next action of the person in question. The structural schematic of our suggested approach is shown in Figure 1.

### 3.1. Pre-Processing of Inertial Sensor Signals

An indispensable first stage of machine learning, image processing, deep learning, and many other areas is image pre-processing. The raw input consists of the videos from which frames are extracted. Subsequently, those frames are very noisy and fuzzy [22]. Due to this, the background of the extracted frames is also very noisy, and hence, the background must be reduced to make individual frames focused on the object of interest, making the quality of the input data useful in subsequent machine learning procedures. It negates necessary background information that could impede the model’s ability to learn the desired features of the target object, thereby increasing its accuracy and the range of its applications. This approach facilitates the ability of the algorithm to pick out pixels belonging to the main object and reject the background features which are not necessarily rich in information but only create clutter. The extraction of the foreground can be explained through Equation (1).

(1) $$M=\sigma \left(W_{conv}^{\left(n\right)}*\sigma \left(\sigma \left(W_{conv}^{\left(1\right)}*I+b_{conv}^{\left(1\right)}\right)\right)+b_{conv}^{\left(1\right)}\right),F=\left(x,y,c\right)=I(x,y,c)\cdot M(x,y)$$

In the equation above, $W_{conv}^{\left(n\right)}$ are the convolutional weight matrices at position (*n*), $b_{conv}^{\left(1\right)}$ are the bias terms added at each convolutional layer, σ denotes the activation function applied element-wise, and M(x,y) represents the value of the mask M at position (x,y). The second layer does not contain the bias, to maintain simplicity, and further, if it is added, the accuracy could easily be combined with the results of the first layer, which already contains the bias. The convolutional weight matrices $W_{conv}$ were trained with the help of gradient-based optimization on a particular dataset. Supervised learning was used to train the model, and if the proper foreground/background data split was absent, then manual labeling or weak supervision is applied to the data. The foreground is obtained by performing element-wise multiplication between the image and the mask, and the areas of interest are highlighted based on the output by the CNN for the particular task. Despite all the operations, the image is still noisy, with many features residing in the edges and corner pixels, and therefore, a blurring filter is used on the image. This is an image processing technique that works to blur the image; that is, it reduces the sharpness of the image, features, or edges in the image. To acquire the pixel values for a smooth image, the values of its neighborhood are taken and averaged. But before that, this process also helps to reduce computational costs and use less information for our system. In the departments of computer vision, image processing, and artificial intelligence, this technique is called the Gaussian blur filter.

Grayscale conversion is a step where the true-color, RGB image is converted to a grayscale image to minimize the workload of 3D images. It is also a well-known pre-processing method for images and is very often used in pattern recognition. It maps each pixel to a shade level between black (0) and white (255) based on the original pixel intensity, effectively converting a three-dimensional picture into a two-dimensional one. This leads to the formation of a single-channel or single-layer image; in this case, these images are relatively simpler to process when compared to the case of RGB images where there are many layers or channels.

**Figure 2:**
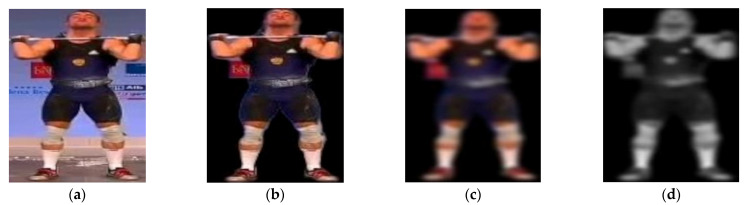
The pre-processing steps are as follows: (**a**) raw frame extracted from a video of Clean & Jerk, (**b**) background noise removed, (**c**) sharpness reduced and edges smoothed, and (**d**) 3D image converted to 2D.

It is clear that how humans perceive color affects the values assigned to the red, green, and blue channels. Figure 2 shows the pre-processing technique step by step.

### 3.2. Human Detection

**Figure 3:**
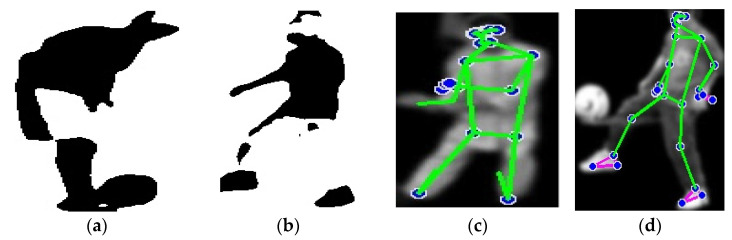
Binary images of humans after silhouette extraction: (**a**) UCF-101; (**b**) YouTube, illustrations of human pose estimation by key points; (**c**) UCF-101; (**d**) YouTube.

In general, human detection entails an understanding of where humans are within a frame or an image. Among the methods that can be used, silhouette extraction takes a full-body image, while another method called pose estimation determines the human body skeleton. To separate the [12,61,62,63] figure from the background, a binary mask is generated, where the pixels belonging to the person are assigned a foreground color (often 1) and all other pixels are assigned a background color (often 0). This step is often critical in most image processing systems. The other typical technique used is applying thresholding to obtain the silhouette from the background. This method sets a threshold value, as in Equation (2), which will give one if the pixel value is higher than it and zero otherwise.

(2) $$S\left(x,y\right)=M(\Theta \left(⎸I\left(x,y\right)-B\left(x,y\right)⎸-\tau \right))$$

where $⎸I\left(x,y\right)-B\left(x,y\right)⎸$ is the key to separating the foreground (human silhouette) from the background by measuring the pixel-wise difference, Θ converts this difference into a binary format, and M refers to the morphological operations that ensure the extracted silhouette is smooth and free of noise. Another approach is pose estimation; it is a technique used to find human joint points. Pose estimation enables performing angle and distance correlations as well as gradient values, which aid in decision-making. It is with this method that we monitor the movement of different joints in the human body [64,65,66,67]. They are usually located in the joints such as the elbow, shoulder, wrist, hip, knee, and ankle and amount to thirty-three points. Equation (3) brings more understanding to this concept. Pose estimation aims to estimate the position as well as the direction of postures of the human body. A common approach to representing human pose estimation mathematically can be represented as

(3) $$P\left(X,Y⎸I\right)=\prod_{i=1}^{N}\left[P_{f}((x_{i},y_{i})⎸I)\cdot \prod_{j\epsilon N(i)}P_{s}(\left(x_{i},y_{i}\right),\left(x_{j},y_{j}\right))\right]$$

where $P\left(X,Y⎸I\right)$ is joint probability distribution of all key point coordinates, $P_{f}((x_{i},y_{i})⎸I)$ is the probability of key point i, and $P_{s}(\left(x_{i},y_{i}\right),\left(x_{j},y_{j}\right)$ is the probability of the spatial relationship between key points i and j, based on their relative positions. The probabilities $P_{f}$ and $P_{s}$ were determined using Gaussian functions (PDF). $P_{f}$ measures how likely a pixel is part of the foreground, while $P_{s}$ accounts for the spatial relationships between neighboring pixels. The parameters of these functions were estimated using training data. In some cases, heatmaps generated by a CNN were used to define $\;P_{f}$, where the intensities in these heatmaps were interpreted as the likelihood of the pixel being part of the foreground. These values were then applied in the calculation of spatial relationships in $P_{s}$. Figure 3 shows the human detection applied to both datasets.

### 3.3. Feature Extraction

**Figure 4:**
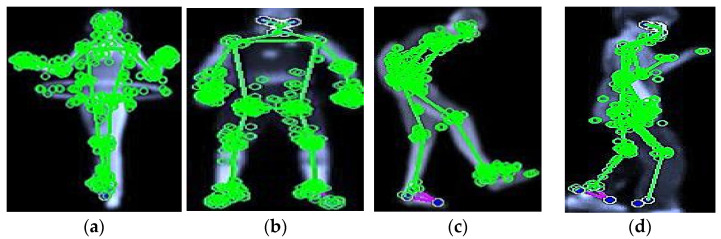
Example of SURF and those circles are showing the extracted keypoints: (**a**,**b**) UCF-101 and (**c**,**d**) YouTube.

Feature extraction is a common step in almost all machine learning and computer vision activities in which raw data are transformed into a form that is easily understandable by a model. The feature extraction [68,69,70] concept entails identifying characteristics or features that are important when it comes to the identification of a conclusion that will make it possible to use the obtained data as an input of the machine learning domain. This process involves recovering features in the image or the frames of the video that might be edges, corners, or texture. In our system, we use six feature extraction techniques: BRIEF, HOG, FAST, and optical flow for each full-body (silhouette) image, and SURF, degree of freedom, distance transform, and MSER for skeleton or joint points. The feature extraction technique known as SURF or Speeded-Up Robust Features is widely used in computer vision. It finds the combination of interest points within an image and calculates the respective descriptors, which helps in recognizing important patterns and textures. Specifically, the SURF is built to be scale- and rotation-invariant, so that the features it finds within the image should not change when the latter is transformed in one way or another. It works in the following way: the method optimizes the approximation of the Hessian matrix for detecting key points, which, despite making the method much faster than SIFT, does not significantly reduce its performance. In practice, SURF finds several interest points by identifying the determinant of the Hessian matrix and then constructing descriptors with the help of the pixel intensity of the local surroundings. Key point detection using the Hessian matrix is explained in Equation (4).

(4) $$F={⋃}_{x\in k}\{d\left(x\right)⎸Det\left(H\left(x,\sigma \right)\right)>\tau ,\theta \left(x\right)={tan}^{-1}(\frac{\sum_{i}w_{i}L_{y}(x_{i})}{\sum_{i}w_{i}L_{x}(x_{i})})\}$$

where k is the set of detected key points in the image, $H\left(x,\sigma \right)$ is Hessian matrix at point x and scale σ, $\theta \left(x\right)$ is the orientation assigned to the key point, and τ is the threshold for key point detection based on the determinant of the Hessian matrix. Figure 4 shows a description of the SURF points.

**Figure 5:**
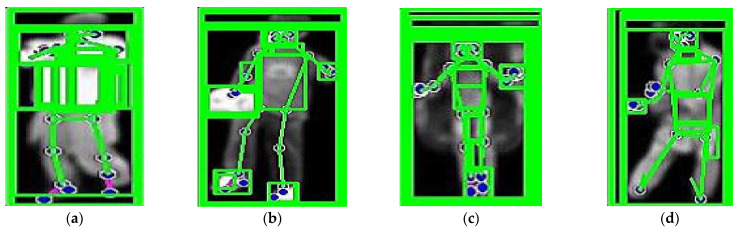
MSER point extraction and the green blocks are showing the stable regions: (**a**,**b**) UCF-101; (**c**,**d**) YouTube.

MSER (Maximally Stable Extremal Regions) is a computer vision algorithm that targets region detection in images with maximum stability under scale changes. First, the basic image intensity is thresholded to obtain a simple binary result; then, the algorithm analyzes the image by combining individual pixels into possible regions. The algorithm then applies stability analysis, exploring how the areas of these regions are changing with shifts in the thresholds, to identify regions that do not change sizes when the thresholds are changed. These stable regions are then selected, coined as extremal regions, making MSER especially useful within applications like object detection and image segmentation, for which there is a need to consistently and accurately detect regions under varying image conditions. In Figure 5, the real-life application of MSER to both datasets is shown. Its mathematical representation is in the following Equation (5):

(5) $$F={⋃}_{k}\left\{C_{t}^{k}⎸∆\left(C_{t}^{k}\right)={min}_{t^{’}\in [t-\in ,t+\in ]}\frac{A\left(C_{t^{’}+\delta }^{k}\right)-A(C_{t^{’}-\delta }^{k})}{A(C_{t^{’}}^{k})}\right\}$$

where ${Cisis}_{t}^{k}$ refers to the connected component (region) in the binary image at threshold t, ${A(C}_{t}^{k})$ is the area (number of pixels) of the connected component, and $∆\left(C_{t}^{k}\right)$ is the stability measure of the connected component.

**Figure 6:**
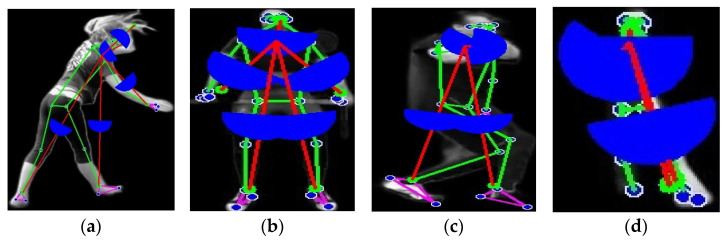
Finding degree points and distances while blue color semi circles are showing the angle between joints: (**a**,**b**) UCF-101; (**c**,**d**) YouTube.

The third approach is the degree of freedom, which strives to measure the freedom at the distance between the points of a joint. This is followed by presenting a new technique for feature extraction, in which, through computer vision and data analytics tools, geometric features are extracted from images of human poses automatically. The joint points are used to identify various geometric characteristics, such as the distances between specific joints (for instance, from the shoulder to the elbow, or between the thigh and the knee, etc.) and some angles formed by a triplet of joints, or other assorted configurations, for example, shoulder–elbow–wrist angles, and so on. It enables an investigator to make a numerical estimate of the body position, motion patterns, and biomechanical traits, hence making it very useful in industries such as biomechanics, sports, physical therapy, and human motion study. The actual computational methodologies that depict the angles are mathematically described in the following Equation (6). Figure 6 is the DoF of a skeleton.

(6) $${DoF}_{total}\left(\lambda \right)=\sum_{i=1}^{r}\frac{\lambda_{i}}{\lambda_{i}+\lambda }-c$$

where r is the rank of the covariance matrix, λ is the regularization parameter in a penalized model, $\lambda_{i}$ is the eigenvalues of the matrix, and c is the number of constraints imposed on the feature set.

**Figure 7:**
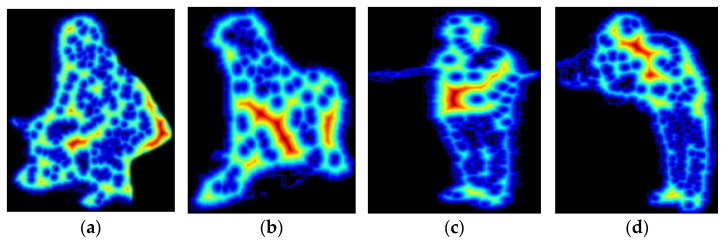
Finding distance transform points and the color scheme is showing intensity: (**a**,**b**) UCF-101; (**c**,**d**) YouTube.

The fourth approach is distance transform. Indeed, distance transform is a fundamental operation in image processing, where a binary image is converted into a distance image, in which the value of each pixel gives the shortest distance from the pixel in question to the nearest pixel in the foreground. Its contribution is useful in many application areas including object segmentation, shape analysis, feature point extraction, and path planning. Consequently, the distance transform is indeed beneficial for enhancing the image processing steps because it determines how far one pixel in an image is from another. To perform distance transformation on the given object, Equation (7) is used.

(7) $$D\left(x,y\right)={min}_{p\in s}(\int_{0}^{1}\sqrt{{\left(\frac{dx\left(t\right)}{dt}\right)}^{2}{+\left(\frac{dy\left(t\right)}{dt}\right)}^{2}}\cdot f(x\left(t\right),y(t))dt$$

where $D\left(x,y\right)$ is the distance function, p∈s is the p  path for set s of all possible paths, $\sqrt{{\left(\frac{dx\left(t\right)}{dt}\right)}^{2}{+\left(\frac{dy\left(t\right)}{dt}\right)}^{2}}$ is the speed or arc, and $f(x\left(t\right),y(t))$ is the function evaluated at $\left(x\left(t\right),y(t)\right)$ pixel values. The output of distance transform can be examined as shown in Figure 7.

**Figure 8:**
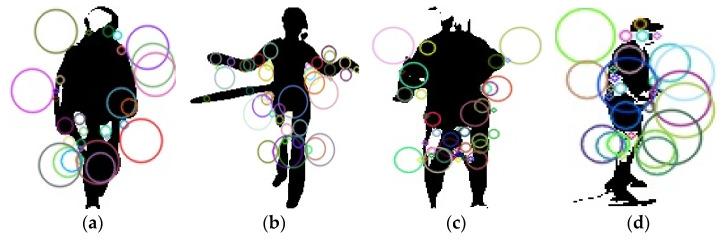
BRIEF feature points: (**a**,**b**) UCF-101; (**c**,**d**) YouTube.

The other three features are BRIEF, HOG, and optical flow, on the entire body (silhouette). Among them, BRIEF, Binary Robust Independent Elementary Features, is one of the most-used feature extraction methods in the computer vision area due to its performance and robustness. In other words, binary descriptors for some of the points that may be marked in an image are obtained. It achieves this by comparing the pixel intensity at certain chosen locations in a local neighborhood around these key points. Surrounding each key point is a window region, known as a patch, which samples pixel pairs with offsets inside this patch. It then examines the relation of the two. If the first pixel’s intensity is more than the second, the value is 1; otherwise, the value is 0. These binary comparisons are summed up to arrive at the binary descriptor of the key point, which can be easily used to search for a similar key point descriptor from some other images. This makes BRIEF particularly suitable for applications where it is required to quickly find the corresponding features for some tasks such as object recognition and tracking. Its implementation can be seen in Equation (8).

(8) $$F_{BRIEF}=\left\{d\left(P_{k}\right)⎸P_{k}\right.=\left\{I\left(x_{k}+u,y_{k}+v\right)⎸\left(u,v\right)\in \left[-\frac{S}{2},\frac{S}{2}\right]\right\},T_{k}\left(I,p_{ki},q_{ki}\right)=\left\{\begin{array}{c} 1\\ 0 \end{array}\right.$$

where $P_{k}$ is the k−th image patch centered at $\left(x_{k},y_{k}\right)$, S is the size of the image, and $T_{k}\left(I,p_{ki},q_{ki}\right)$ is the binary test result, which is 1 if the intensity at $p_{i},$ is less than the intensity at $q_{i}$, and 0 otherwise. The examples of both datasets can be seen in Figure 8.

**Figure 9:**
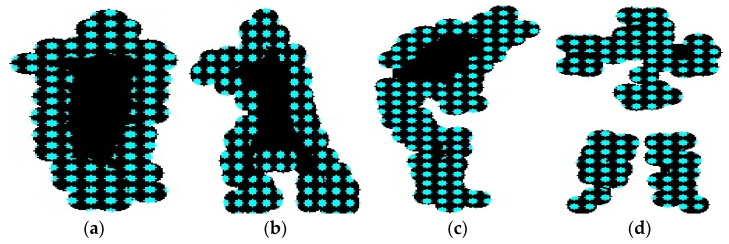
ORB features extracted: (**a**,**b**) UCF-101; (**c**,**d**) YouTube.

Contour tracking in the field of computer vision plays a significant role and is especially critical in calculating optical flow when studying a person’s motion between frames. This is especially useful when one wants to follow a feature across different frames of a video or across different images. In this process, the widely used feature extraction technique known as the Lucas–Kanade method is incorporated with the calculation of the optical flow. First of all, it defines the features to track, followed by the estimation of the intensity gradient in the second step, and in the final step, the solution of a set of linear equations for the motion parameters is given. It is vital in numerous computer vision applications such as moving object tracking, moving object analysis, and video stabilization. To further clarify this concept, let us take a look at Equation (9) and Figure 9.

(9) v=−(∑i∈W[Ixi2IxiIyiIxiIyiIyi2])−1∑i∈W[IxiItiIyiIti]

where *v* represents the velocity vector we want to calculate. $I_{x},I_{y},\;and\;I_{t}$ represent the partial derivatives of the image intensity I with respect to the x−axis, y−axis, and time t, respectively. The summation is performed over a window W of pixels. The first term is a 2 × 2 matrix of second-order derivatives. The second term is a 2 × 1 vector involving the temporal derivative.

**Figure 10:**
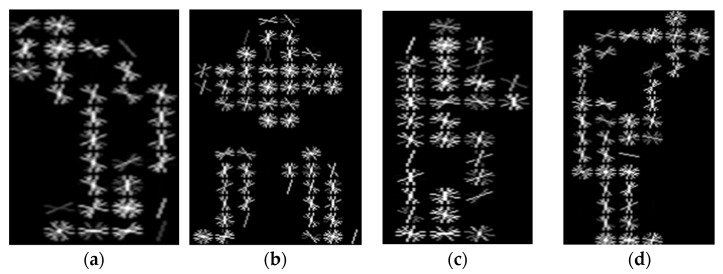
HOG gradient points: (**a**,**b**) UCF-101; (**c**,**d**) YouTube.

HOG, or Histogram of Oriented Gradients, is a feature description technique that is often used in image processing, especially for detection/recognition [29]. It operates based on the assumption that in small parts of an image, the gradients’ directions and magnitudes need to be evaluated. These orientations are then used to build histograms inside a small cell, and in the block, the values [71,72,73] are normalized to increase stability. All these normalized histograms are concatenated together to sum up a detailed textural and edge relationship, which is described in the figure below. HOG is good, especially in describing local image patterns, which makes it useful in the areas that require the capturing of detailed images, such as in the cases of analyzing cars, people in the pictures, objects, and even scenes in a landscape or any other similar activity. The above process is further described in Equation (10) and is presented in the framework represented in Figure 10.

(10) H=[∑(x,y)∈b1…nM(x,y)·δ(b−bθx,y)Hb1…n22+ϵ2]

where M(x,y) is the gradient magnitude, $\theta \left(x,y\right)$ is the gradient orientation, $b\left(\theta \right)$ is the orientation bin for the angle $\theta \left(x,y\right),$ and $\delta (b-b\left(\theta \right)$ is an indicator function that assigns pixel contributions to the correct histogram bin.

**Figure 11:**
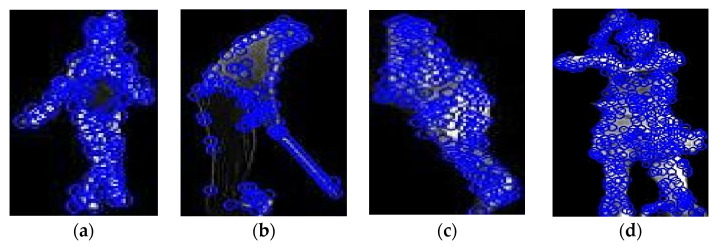
FAST feature points: (**a**,**b**) UCF-101; (**c**,**d**) YouTube.

The last feature is FAST. The process known as FAST, or “Features from Accelerated Segment Test,” is used to identify a digital image’s key points. It is extensively utilized in computer vision applications because it was made for effective and real-time feature detection. The “corner” pixel in the image is the first pixel selected as a possible key point. The program sets a threshold value and then measures the intensity of this corner pixel by comparing it with the intensities of the 16 surrounding pixels, which are arranged in a circle. It is regarded as a possible key point if at least 12 of these surrounding pixels, measured by the threshold value, are either darker or brighter than the corner pixel, as shown in Figure 11. The corner pixels are extracted by the following Equations (11) and (12).

(11) $$P\left(C\left(p\right)\right)=\frac{\sum_{p}C(P)}{\sum_{i=1}^{16}\delta (c_{i})}$$

where

(12) $$\delta \left(c_{i}\right)=\left\{\begin{array}{c} 1,\;I\left(c_{i}\right)>I\left(p\right)=t\;or\;I\left(c_{i}\right)<I\left(p\right)-t\\ 0,\;Otherwise\; \end{array}\right.$$

where $P\left(C\left(p\right)\right)$ is the corner probability, $C\left(p\right)$ is the corner criterion, $\delta \left(c_{i}\right)$ is the indicator function, $I\left(c_{i}\right)$ is the intensity of indicator pixels, $I\left(p\right)$ is the intensity of candidate pixel, and t is the threshold value.

### 3.4. Features Fusion

Another concept we come across is known as feature fusion, where features originating from various sources or sensors, modalities, and various algorithms are combined to improve tasks such as classification, clustering, and optimization. Early or feature-level fusion, which combines data in their raw or modified form at the initial stage of the processing stream, is utilized in the proposed approach. This creates a continuous feature vector that combines information from several inputs, creating better data representation.

In order to make the early fusion effective, we conduct a correlation and dependency analysis of the selected features. Some reasons are given in this analysis in support of the choice of descriptors that we choose and the impact they have on the model. For instance, it is possible to determine the relationships of features by using correlation coefficients or even mutual information. This is flexible enough to enable the selection of features that not only improve the general data representation but also improve performance, particularly in subsequent tasks.

**Figure 12:**
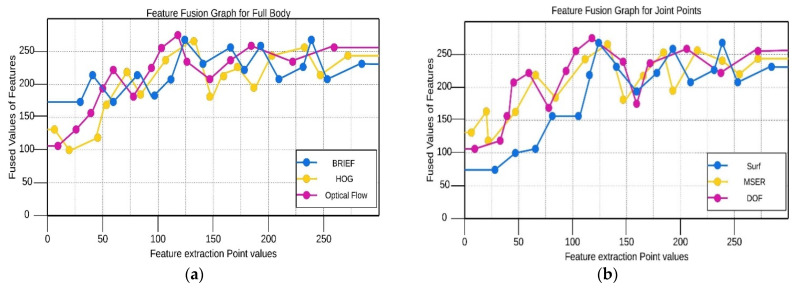
Feature fusion graphical representation: (**a**) full-body points (silhouettes); (**b**) pose estimation points.

Early fusion approaches commonly simplify the model architecture by combining all input features into a single vector, thus making the model ready to process data in a single form. This approach models dependencies between features and combines different data types effectively while preserving simplicity and requiring no multiple heterogeneous data streams. This, in a way, creates a more integrated model that enhances the performance of pattern matching and forecasting. For a better understanding, we will use Equation (13). Rocchio’s algorithm can also be applied to merge the vectors of the same feature spaces into a single vector. Figure 12 is the comparison of graphs of both human detection techniques.

(13) $$q_{m}=\alpha q_{o}+\beta_{1}|I_{r}|\sum_{ij\in I_{r}}I_{j}ij-\gamma_{1}|I_{nr}|\sum_{ij\in I_{nr}}I_{j}$$

In this equation, $q_{m}$ represents the modified query, $q_{o}$ is the original query, $I_{r}$ denotes the collection of relevant documents or images, and $I_{nr}$ is the collection of non-relevant documents. The weights are denoted by $\alpha ,{\;\beta }_{1}$, and $\gamma_{1}$.

### 3.5. Optimization

**Figure 13:**
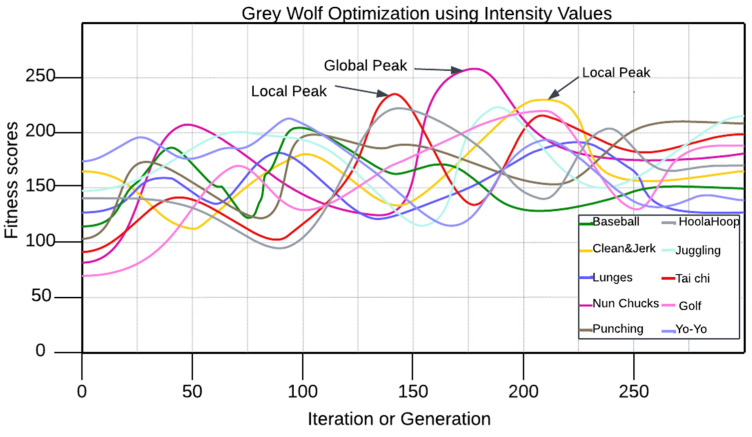
Flow graph of Grey Wolf Optimization points.

The concept of feature optimization in the realms of machine learning and data analysis entails arriving at the best features in the dataset [74,75,76,77]. The Grey Wolf Optimization algorithm (GWO) is a metaheuristic optimization algorithm based on gray wolves’ social structure, scheming, and hunting mechanisms. In this algorithm, the population is split into the alpha, beta, delta, and omega populations, where the alpha population is the dominant population, and the omega population is lowest-ranking population. The studies show that the alphas hunt, and the rest of the breeding pack assists the alphas in hunting in precise coordination. The position of each wolf is updated by the algorithm determined by the best solutions in the iteration cycle; these steps correspond to the encircling, hunting, and attacking modes of a real wolf pack. GWO is employed as a solver for optimization problems because of the algorithm’s simplicity and flexibility, and the absence of the local optimum problem in solving the optimization problems, as explained in Equation (14).

(14) $$X_{i}\left(t+1\right)=\frac{1}{3}(X_{\alpha }\left(t\right)-A_{\alpha }\cdot D_{\alpha }+X_{\beta }\left(t\right)-A_{\beta }\cdot D_{\beta }+X_{\delta }\left(t\right)-A_{\delta }\cdot D_{\delta })$$

where $X_{i}(t+1)$ refers to the updated position of wolf i at iteration (t+1), $X_{\alpha }\left(t\right),X_{\beta }\left(t\right)$ ,and $X_{\delta }\left(t\right)$ refer to positions of the best three wolves at iteration t, $A_{\alpha },A_{\beta }$ ,and $A_{\delta }$ refer to coefficients that adjust the influence of the best wolves based on random factors and a linearly decreasing component *a*, and $D_{\alpha },D_{\beta }$ ,and $D_{\delta }$ refer to distances between the current wolf’s position and the positions of the best wolves. A depiction of Grey Wolf Optimization is shown in Figure 13.

### 3.6. Event Classifier: CNN + RNN Hybrid Classifier

For tasks where temporal and spatial inputs are required, like video analysis and action identification, the integration of CNN and RNN has been quite successful. CNNs employ convolutions that capture features such as edges, form, and texture, which are important for identifying patterns and capturing spatial properties. CNNs by themselves, however, cannot reproduce the time series of event sequences, which is an important requirement for tasks involving sequences.

**Figure 14:**
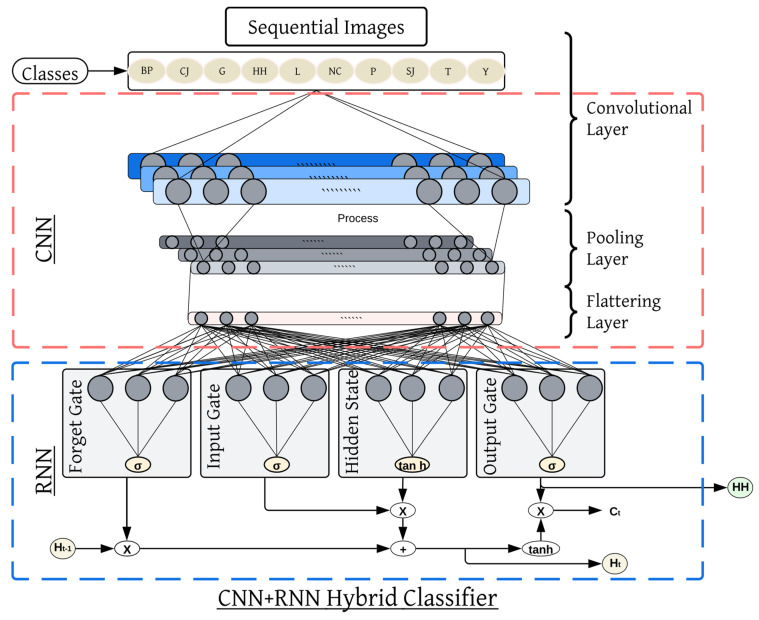
Exemplary structure of hybrid classifier.

In the second case where the elements are ordered, Recurrent Neural Networks, especially Long Short-Term Memory (LSTM) and Gated Recurrent Units (GRUs), are extremely useful. RNNs are capable of maintaining some information from the previous steps to understand the sequence of the occurrence and hence can be used to handle sequential data. Similar to the initial processing of individual video frames, in the hybrid model of CNN-RNN, feature vectors representing spatial information for each frame are generated. The RNN then feeds these vectors by tracking changes as a sequence of the frames to look for temporal patterns. For tasks such as recognizing programs associated with sporting activities or describing events occurring in videos, this technique allows the model to learn not only what is going on frame by frame but also how actions transform in time. One of the main features of the design is the ability to blend spatial and temporal organization, making it highly feasible for controlling complex data over time. Figure 14 is the functional structure of our proposed hybrid classifier. Algorithm 1 shows the basic functional architecture of this hybrid classifier.
**Algorithm 1:** CNN + RNN Hybrid Classifier for sports and event classification.**Input:** Sequence of video frames $\;X=\left\{x_{1}\right.,{\;x}_{2},\;\;x_{3},\;\cdot \cdot \cdot \cdot \cdot ,\left.{\;x}_{T}\right\}$ **Output:** Predicted class label  ŷ **(1) Initialize the following:**   • Set initial hidden state $h_{0}$ and cell state $C_{0}$ for RNN (e.g., LSTM).**(2) For each frame** $x_{t}$ **in the sequence X, perform the following:**   • **Step 1: Extract spatial features with CNN**        • Compute feature vector $f_{t}$ by applying the CNN to frame $x_{t}$:
$f_{t}=CNN(x_{t})$
   • **Step 2: Update temporal state with RNN**
      • Update the RNN hidden state and cell state using the following equations:
$i_{t}=\sigma (W_{i}f_{t}+U_{i}h_{t-1}+b_{i}$ (input gate) $f_{t}=\sigma (W_{f}f_{t}+U_{f}h_{t-1}+b_{f}$ (forget gate) ${Ĉ}_{t}=\;tanh(W_{c}f_{t}+U_{c}h_{t-1}+b_{c}$ (cell candidate) $C_{t}=f_{t}\cdot C_{t-1}+i_{t}\cdot {Ĉ}_{t}$ (cell state) $0_{t}=\sigma (W_{0}f_{t}+U_{0}h_{t-1}+b_{0})$ (output gate) $h_{t\;}={\;0}_{t}\cdot tanh(C_{t})$ (hidden state)**(3) Final classification**
  • After processing all frames, compute the output prediction ŷ using the final hidden state $h_{T}$.
$ŷ=softmax\;W_{y}\cdot h_{t}+b_{y}$**Output:** Return the predicted class label
 ŷ

## 4. Dataset Experimental Setup and Results

Here, we provide the results section of the demonstrated approach and compare it to other works addressing the same issue. Positive outcomes with the assessment of these datasets can be highlighted [78,79,80] in terms of the accuracy achieved when compared to prior studies.

For our research, we utilized two widely recognized benchmark datasets in the field of action recognition. The dataset used in this experiment is UCF-101, and the second one is the YouTube dataset. The UCF-101 is a dataset formed by 13,320 clips divided into 101 action classes that include various types of activities including sports and daily-life events. The YouTube dataset has 8 million videos and more than 3700 classes. The UCF-101 dataset, widely used in action recognition research, provides a predefined split, with 9537 videos used for training and 3783 for testing. In our experiments, we used this standard division. For the YouTube dataset, however, we performed cross-validation for the testing/training sets. For additional validation, we employed n-fold cross-validation on the YouTube dataset to ensure robustness. It is also worth mentioning that no transfer learning approach was applied in this work; all models from all the datasets were learned from scratch.

The experiments were carried out in Python on a system with the Intel Core i5 processor and 8GB RAM. The effectiveness of the recognition model shall be analyzed with accuracy, defined in Equation (15); precision, defined in Equation (16); and recall, defined in Equation (17). The results showed that the proposed approach achieved 98.5% on UCF-101 and 99.2% on YouTube datasets. These confusion matrices are shown in Table 1 and Table 2, and the comparison per class concerning precision, accuracy, and recall is shown in Table 3 and Table 4. Table 5 shows the performance metrics of the proposed system including predicted accuracy, error margin, and confidence level. The final comparative summary is given in Table 6. In this paper, we evaluated 10 classes from both datasets. The proposed approach processes each frame in approximately 27.5 ms, combining steps such as pre-processing (~2 ms), silhouette and pose estimation (~5 ms), feature extraction (~10 ms), dimensionality reduction (~0.5 ms), and classification (~10 ms). This results in an estimated speed of about 36.36 frames per second (fps), demonstrating the method’s efficiency in handling the entire action recognition pipeline; when calculating practically, the speed characteristic can easily reach 30 frames per second. While comparing with other research, our speed characteristic is good and efficient.

(15) $$Accuracy=\frac{\left(TP+TN\right)}{\left(TP+FP+TN+FN\right)}$$

(16) $$Precision=\frac{TP}{\left(TP+FP\right)}$$

(17) $$Recall=\frac{TP}{\left(TP+FN\right)}$$

In the above equations, TP stands for True Positives—the positives that have been accurately predicted; TN stands for True Negatives—the negatives that have been accurately predicted; FP represents False Positives—the number of times predictions made as positives are actually negatives; and FN represents False Negatives—the number of times predictions made as negatives are actually positives.

In our study, the proposed model emphasizes investigating the algorithms of the machine learning approaches. Some of the cited studies in the following table obtained slightly higher accuracies than our proposed model; this is because they employed deep learning approaches. As for future work, we plan to merge various types of deep learning approaches over several steps like segmentation [81], feature extraction, and classification level. Based on certain observations, we believe that our model will enhance the accuracy factor.

**Table 5:** Performance metrics of the proposed system.

UCF-101 Dataset		YouTube Dataset	
**Metric**	**Value**	**Metric**	**Value**
**Predicted Accuracy**	98.00–99.00%	**Predicted Accuracy**	98.83–99.57%
**Error Margin**	±0.50%	**Error Margin**	±0.37%
**Confidence Level**	95%	**Confidence Level**	95%

**Table 6:** Comparison of the proposed model with state-of-the-art methods.

State-of-the-Art Systems	UCF-101(%)	State-of-the-Art Systems	YouTube (%)
Akhter et al. [82]	82.6	Meng et al. [83]	82.5
Tayyab et al. [60]	84.7	De Siva et al. [84]	84.2
Almeida et al. [85]	93.9	Akhter et al. [86]	85.0
Abdulazeem et al. [87]	94.8	-	-
Shrestha et al. [88]	94.8	-	-
**Proposed System**	**98.5**	**Proposed System**	**99.2**

For better analysis of the performance of our proposed system, Table 7 is added, which is the ablation table, which clarifies the system performance at each step. The Adaboost classifier is used for comparison purposes so that the performance can be easily understood.

## 5. Failure Cases

During the pre-processing stage, the background of the image is removed to better focus on the subject in the frame. However, this process tends to create foreground residual noise, which remains unprocessed on many occasions. This residual noise normally happens because noisy areas are composed of linked pixels, which the system misinterprets as belonging to the principal figure. For example, certain object shapes, shading, or even other objects in the scene may resemble the target object and hence cause misclassification.

Such inaccuracies introduce ambiguity into the segmentation process, and it becomes difficult to separate the main subject. This, in turn, distorts the subsequent processes of feature extraction because the features extracted contain information that is either irrelevant or misleading. Such a problem is solved with the help of human detection algorithms, though it does not affect the overall accuracy of the system, because the high performance of human detection ensures accurate identification of the subject. In other words, misclassified noise on one level is balanced out by human detection on another, where the main contour of the target object is sought as opposed to segmentation at the pixel level.

**Figure 15:**
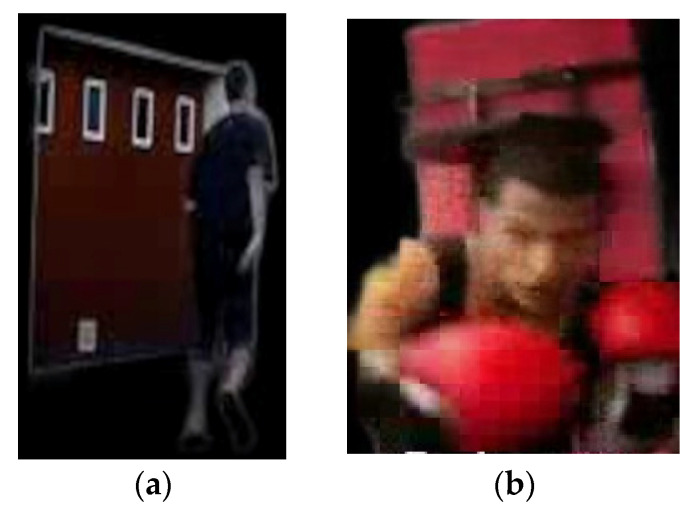
Failure cases of background removal during our experiments: (**a**) basketball dunk; (**b**) boxing.

Nonetheless, it should be noted that compensation remains partial, and still, some limitations are observed. These are especially apparent in motion-related or multifaceted activities, like boxing and basketball dunks, as they include quick motion and obfuscate structures that raise the stairway of failure cases. For instance, it may be difficult for the system to distinguish, for example, the player’s arm from the crowd during the dunk sequence of basketball or parts of the punching bag as noise. Hence, these failure cases happen in some frames of the mentioned classes that introduce more noise, thereby emphasizing the need for better pre-processing for image segmentation. Some of the examples of such system failures are explained diagrammatically in Figure 15.

## 6. Conclusions and Future Work

The proposed method in this research unifies various approaches toward feature extraction and tunable classifiers for major improvements in event detection and behavior analysis. As feature descriptors, four novel combinations of feature descriptors used in the proposed approach include MSER, SURF, distance transform, and DOF of the skeleton points; BRIEF, HOG, FAST, and Optical Flow of the silhouettes; or full-body points. This work, when complemented with Grey Wolf Optimization and a Hybrid CNN + RNN classifier, delivers near-optimal performance on the UCF-101 dataset of 98.5% and on the YouTube dataset of 99.2%, thus outperforming existing approaches. The findings confirm the strength of the method when it comes to solving problems like variability in the conditions it has to operate under, and the speed, which makes it suitable for use in real-life situations. Appropriate application areas include surveillance technologies, sports performance, and behavior-tracking applications. This work not only presented one of the best results for the present recognition of human actions but also opened a door for future research for scenes with a large number of humans in them. Future directions are to increase the number of features, use unsupervised learning to extract them, expand the method to analyze dynamic processes (drone video, crowds in public spaces), and enhance the algorithm in cases of multi-person scenarios.

## Figures and Tables

**Table 1:** Confusion matrix of the UCF-101 dataset.

Proposed HAR	Bb	Cj	G	Hh	L	Nc	P	Sj	T	Y
Bb	**98**	0	1	0	0	0	1	0	0	0
Cj	0	**99**	0	0	0	0	0	1	0	0
G	0	0	**98**	1	0	0	1	0	0	0
Hh	0	0	0	**100**	0	0	0	0	0	0
L	0	0	1	1	**97**	0	0	0	0	1
Nc	0	0	0	0	0	**99**	0	0	0	1
P	0	0	0	0	0	1	**99**	0	0	0
Sj	0	0	0	1	0	0	1	**98**	0	0
T	0	0	0	1	0	0	0	0	**98**	1
**Y**	0	1	0	0	0	0	0	0	0	**99**
**Mean Accuracy = 98.5%**										

Bb = Baseball; Cj = Clean & Jerk; G = Golf; Hh = Hula-hoops; L = Lunges; Nc = Nun Chucks; P = Punching; S = Soccer; T = Taiichi; Y = Yo Yo.

**Table 2:** Confusion matrix of the YouTube dataset.

Proposed HAR	Bb	Bp	D	G	Hr	J	S	Sj	T	W
Bb	**99**	0	1	0	0	0	0	0	0	0
Bp	0	**99**	0	0	1	0	0	0	0	0
D	0	0	**99**	1	0	0	0	0	0	0
G	0	0	1	**98**	0	0	0	1	0	0
Hr	0	0	0	0	**100**	0	0	0	0	0
J	0	0	0	1	0	**99**	0	0	0	0
S	0	0	0	0	0	0	**99**	0	0	1
Sj	0	0	0	0	0	0	0	**100**	0	0
T	0	0	0	0	0	0	0	0	**99**	1
W	0	0	0	0	0	0	0	0	0	**100**
**Mean Accuracy = 99.2%**										

Bb = Baseball; Bp = Baseball pitch; D = Diving; G = Golf; Hr = Horse riding; J = Jumping; S = Swing; Sj = Soccer Juggling; T = Tennis; W = Walking.

**Table 3:** Class-wise precision, recall, and accuracy breakdown for the UCF-101 dataset.

Events	Accuracy	Precision	Recall
**Baseball Pitch**	0.998	1.000	0.980
**Clean & Jerk**	0.998	0.990	0.990
**Golf**	0.996	0.980	0.980
**Hula Hoops**	0.996	0.962	1.000
**Lunges**	0.997	1.000	0.970
**Nun Chucks**	0.998	0.990	0.990
**Punching**	0.996	0.971	0.990
**Soccer Juggling**	0.997	0.990	0.980
**Taiichi**	0.998	1.000	0.980
**YoYo**	0.996	0.971	0.990
**Mean**	**0.997**	**0.985**	**0.985**

**Table 4:** Class-wise precision, recall, and accuracy breakdown for the YouTube dataset.

Events	Accuracy	Precision	Recall
**Basketball**	0.999	1.000	0.990
**Baseball**	0.999	1.000	0.990
**Diving**	0.997	0.980	0.990
**Golf**	0.996	0.980	0.980
**Juggle**	0.999	0.990	1.000
**Horse riding**	0.999	1.000	0.990
**Jumping**	0.999	1.000	0.990
**Swing**	0.999	0.990	1.000
**Tennis**	0.999	1.000	0.990
**Walking**	0.998	0.980	1.000
**Mean**	**0.998**	**0.992**	**0.992**

**Table 7:** An ablation experiment evaluating all methods on both datasets.

Ablation Settings	Pre-Processing	MSER + SURF + DOF (Skeleton)	BRIEF + HOG + Optical Flow (Silhouette)	FAST	Distance Transform	Early Fusion	Grey Wolf Optimization	AdaBoost Classifier	Hybrid Classifier	Accuracy	
										UCF-101	YouTube
**Baseline**	✓	✗	✗	✗	✗	✗	✗	✓	✗	68.4%	69.2%
**Skeleton features only**	✓	✓	✗	✗	✗	✗	✗	✓	✗	73.4%	74.2%
**Silhouette feature only**	✓	✗	✓	✗	✗	✗	✗	✓	✗	78.6%	79.5%
**Skeleton + silhouette feature**	✓	✓	✓	✗	✗	✗	✗	✓	✗	80.2%	81.1%
**+ feature fusion**	✓	✓	✓	✗	✗	✓	✗	✓	✗	85.7%	86.3%
**+ feature fusion + optimizer**	✓	✓	✓	✗	✗	✓	✓	✓	✗	97.9%	88.6%
**FAST + DT with other classifiers**	✓	✓	✓	✓	✓	✓	✓	✓	✗	89.3%	90.1%
**Other classifiers without FAST + DT**	✓	✓	✓	✗	✗	✓	✓	✓	✗	91.2%	92.6%
**Full system with Hybrid Classifier**	✓	✓	✓	✓	✓	✓	✓	✗	✓	**98.5%**	**99.2%**

The green color tick mark shows that algorithm is used and red color cross means the algorithm was not used.

## Data Availability

Both datasets are publicly available on kaggle.

## References

[1] Tiwary A., Dhidhi J., Gupta V.K., Mandi B.C. Object Detection and Land-marking for Human Action Recognition in Single to Multiplayer Sports by Hybrid Approach. Proceedings of the 2024 15th International Conference on Computing Communication and Networking Technologies (ICCCNT).

[2] Pagare S., Kumar R. (2024). Human Action Recognition using Long Short-Term Memory and Convolutional Neural Network Model. Int. J. Soft Comput. Eng..

[3] Kulsoom F., Narejo S., Mehmood Z., Bashir A.K., Mehmood Z., Chaudhry H.N. (2022). A Review of Machine Learning-based Human Activity Recognition for Diverse Applications. Neural Comput. Appl..

[4] Dang D.M.N., Duong V.H., Wang J.C., Duc N.B. (2024). YOWOv3: An Efficient and Generalized Framework for Human Action Detection and Recognition. arXiv.

[5] Janani P., Suratgar A., Taghvaeipour A. (2024). Enhancing Human Action Recognition and Violence Detection Through Deep Learning Audiovisual Fusion. arXiv.

[6] Alhakbani N., Alghamdi M., Al-Nafjan A. (2023). Design and Development of an Imitation Detection System for Human Action Recognition Using Deep Learning. Sensors.

[7] Li Y. (2024). Research on Human Action Recognition Method Based on Machine Learning. Highlights Sci. Eng. Technol..

[8] Yu Z., Yan W.Q. Human Action Recognition Using Deep Learning Methods. Proceedings of the 2020 35th International Conference on Image and Vision Computing New Zealand (IVCNZ).

[9] Yang L., Hao Y., Shinoda K. Sensor Data Representation with Transformer-Based Contrastive Learning for Human Action Recognition and Detection. Proceedings of the 2023 31st European Signal Processing Conference (EUSIPCO).

[10] Chopra H., Mundody S., Guddeti R.M.R. A Key-frame Extraction for Object Detection and Human Action Recognition in Soccer Game Videos. Proceedings of the 2023 14th International Conference on Computing Communication and Networking Technologies (ICCCNT).

[11] Manakitsa M.N., Maraslidis G.S., Moysis L., Fragulis G.F. (2024). A Review of Machine Learning and Deep Learning for Object Detection, Semantic Segmentation, and Human Action Recognition in Machine and Robotic Vision. Technologies.

[12] Nadeem A., Jalal A., Kim K. (2020). Accurate Physical Activity Recognition using Multidimensional Features and Markov Model for Smart Health Fitness. Symmetry.

[13] Al Mudawi N., Ansar H., Alazeb A., Aljuaid H., AlQahtani Y., Algarni A., Jalal A., Liu H. (2024). Innovative Healthcare Solutions: Robust Hand Gesture Recognition of Daily Life Routines Using 1D CNN. Front. Bioeng. Biotechnol..

[14] Awan A.A., Najam S., Jalal A. Robust Exercise-Based Telerehabilitation for Elderly Healthcare Services. Proceedings of the 2024 19th International Conference on Emerging Technologies (ICET).

[15] Afsar M.M., Saqib S., Ghadi Y.Y., Alsuhibany S.A., Jalal A., Park J. (2022). Body Worn Sensors for Health Gaming and E-Learning in Virtual Reality. Comput. Mater. Contin..

[16] Liu J., Luo J., Shah M. Recognizing Realistic Actions from Videos “in the Wild”. Proceedings of the 2009 IEEE Conference on Computer Vision and Pattern Recognition (CVPR).

[17] Khan M.A., Javed K., Khan S.A., Saba T., Habib U., Khan J.A., Abbasi A.A. (2020). Human Action Recognition Using Fusion of Multiview and Deep Features: An Application to Video Surveillance. Multimed. Tools Appl..

[18] Kamal S., Jalal A. A Novel Human Interaction Recognition via Composite Features and Max Entropy Classifier. Proceedings of the 2024 19th International Conference on Emerging Technologies (ICET).

[19] Kamal S., Jalal A. Multi-Feature Descriptors for Human Interaction Recognition in Outdoor Environments. Proceedings of the 2024 International Conference on Engineering & Computing Technologies (ICECT).

[20] Bay H., Tuytelaars T., Van Gool L. (2006). Surf: Speeded Up Robust Features. Proceedings of the Computer Vision–ECCV 2006: 9th European Conference on Computer Vision.

[21] Calonder M., Lepetit V., Strecha C., Fua P. Brief: Binary Robust Independent Elementary Features. Proceedings of the Computer Vision–ECCV 2010: 11th European Conference on Computer Vision.

[22] Matas J., Chum O., Urban M., Pajdla T. (2004). Robust Wide-Baseline Stereo from Maximally Stable Extremal Regions. Image Vis. Comput..

[23] Dalal N., Triggs B. Histograms of Oriented Gradients for Human Detection. Proceedings of the 2005 IEEE Computer Society Conference on Computer Vision and Pattern Recognition (CVPR’05).

[24] Rachmadi R., Uchimura K., Koutaki G. Combined Convolutional Neural Network for Event Recognition. Proceedings of the Korea-Japan Joint Workshop on Frontiers of Computer Vision.

[25] Paul A., Mukherjee D.P., Das P., Gangopadhyay A., Chintha A.R., Kundu S. (2018). Improved Random Forest for Classification. IEEE Trans. Image Process..

[26] Resende PA A., Drummond A.C. (2018). A Survey of Random Forest Based Methods for Intrusion Detection Systems. ACM Comput. Surv..

[27] Huang S., Cai N., Pacheco P.P., Narrandes S., Wang Y., Xu W. (2018). Applications of Support Vector Machine (SVM) Learning in Cancer Genomics. Cancer Genom. Proteom..

[28] Tran D.N., Phan D.D. Human Activities Recognition in Android Smartphone Using Support Vector Machine. Proceedings of the 2016 7th International Conference on Intelligent Systems, Modelling and Simulation (ISMS).

[29] O’Shea K., Nash R. (2015). An Introduction to Convolutional Neural Networks. arXiv.

[30] Li Z., Liu F., Yang W., Peng S., Zhou J. (2021). A Survey of Convolutional Neural Networks: Analysis, Applications, and Prospects. IEEE Trans. Neural Netw. Learn. Syst..

[31] Shi Y., Xi J., Hu D., Cai Z., Xu K. (2023). RayMVSNet++: Learning Ray-Based 1D Implicit Fields for Accurate Multi-View Stereo. IEEE Trans. Pattern Anal. Mach. Intell..

[32] Tayyab M., Jalal A. A Novel Sports Event Recognition using Pose Estimation and MultiFused Features. Proceedings of the 2024 International Conference on ETECTE.

[33] He S., Luo H., Jiang W., Jiang X., Ding H. (2024). VGSG: Vision-Guided Semantic-Group Network for Text-Based Person Search. IEEE Trans. Image Process..

[34] Zheng C., An Y., Wang Z., Wu H., Qin X., Eynard B., Zhang Y. (2022). Hybrid Offline Programming Method for Robotic Welding Systems. Robot. Comput.-Integr. Manuf..

[35] Tayyab M., Jalal A. Advanced Gait Event Recognition and Pose Estimation Model through Deep Learning. Proceedings of the 2024 International Conference on IT and Industrial Technologies (ICIT).

[36] Sun J., Zhou L., Geng B., Zhang Y., Li Y. (2024). Leg State Estimation for Quadruped Robot by Using Probabilistic Model with Proprioceptive Feedback. IEEE/ASME Trans. Mechatron..

[37] Wang Y., Chen H., Law J., Du X., Yu J. (2023). Ultrafast Miniature Robotic Swimmers with Upstream Motility. Cyborg Bionic Syst..

[38] Pervaiz M., Shorfuzzaman M., Alsufyani A., Jalal A., Alsuhibany S.A., Park J. (2023). Tracking and Analysis of Pedestrian’s Behavior in Public Places. Comput. Mater. Contin..

[39] Alarfaj M., Pervaiz M., Ghadi Y.Y., Al Shloul T., Alsuhibany S.A., Jalal A., Park J. (2023). Automatic Anomaly Monitoring in Public Surveillance Areas. Intell. Autom. Soft Comput..

[40] Khan D., Al Mudawi N., Abdelhaq M., Alazeb A., Alotaibi S.S., Algarni A., Jalal A. (2024). A Wearable Inertial Sensor Approach for Locomotion and Localization Recognition on Physical Activity. Sensors.

[41] Bukht T.F.N., Al Mudawi N., Alotaibi S.S., Alazeb A., Alonazi M., AlArfaj A.A., Jalal A., Kim J. (2023). A Novel Human Interaction Framework Using Quadratic Discriminant Analysis with HMM. Comput Mater. Contin..

[42] Alzubaidi L., Zhang J., Humaidi A.J., Al-Dujaili A., Duan Y., Al-Shamma O., Santamaría J., Fadhel M.A., Al-Amidie M., Farhan L. (2021). Review of Deep Learning: Concepts, CNN Architectures, Challenges, Applications, Future Directions. J. Big Data.

[43] Alabdullah B.I., Ansar H., Mudawi N.A., Alazeb A., Alshahrani A., Alotaibi S.S., Jalal A. (2023). Smart Home Automation Based Hand Gesture Recognition Using Feature Fusion and RNN. Sensors.

[44] Mushhood M., Shizza S., Aladfaj M., Hamad M., Khaled A., Hanan A., Jalal A., Park J. (2023). Body-Worn Sensors for Recognizing Physical Sports Activities in Exergaming via Deep Learning Model. IEEE Access.

[45] Alazeb A., Bisma C., Naif Al M., Yahya A., Alonazi M., Hanan A., Ahmad J., Hui L. (2024). Remote Intelligent Perception System for Multi-Objects Detection. Front. Neurorobotics.

[46] Abbas Y., Jalal A. Drone-Based Video Surveillance Using YOLOv6 and Neuro-Fuzzy Classifier. Proceedings of the 2024 19th International Conference on Emerging Technologies (ICET).

[47] Abbas Y., Jalal A. Drone-Based Human Action Recognition for Surveillance: A Multi-Feature Approach. Proceedings of the International Conference on Engineering & Computing Technologies (ICECT).

[48] Sohan M., Ram T., Ch V. (2024). A Review on YOLOv8 and Its Advancements. Advancements in Artificial Intelligence and Machine Learning.

[49] Raza A., Chelloug S.A., Alatiyyah M.H., Jalal A., Park J. (2023). Multiple Pedestrian Detection and Tracking in Night Vision Surveillance Systems. Comput. Mater. Contin..

[50] Azmat U., Alotaibi S.S., Abdelhaq M., Alsufyani N., Shorfuzzaman M., Jalal A., Park J. (2023). Aerial Insights: Deep Learning-Based Human Action Recognition in Drone Imagery. IEEE Access.

[51] Azmat U., Alotaibi S.S., Al Mudawi N., Alabduallah B.I., Alonazi M., Jalal A., Park J. (2023). An Elliptical Modeling Supported System for Human Action Deep Recognition over Aerial Surveillance. IEEE Access.

[52] Mahwish P., Ahmad J. Artificial Neural Network for Human Object Interaction System over Aerial Images. Proceedings of the 2023 4th International Conference on Advancements in Computational Sciences (ICACS).

[53] Shrestha P., Pandey S. (2023). Human Action Recognition Using Deep Learning Methods. Proceedings of the International Conference on Machine Learning and Data Engineering.

[54] Muneeb M., Hammad R., Jalal A. Automate Appliances via Gestures Recognition for Elderly Living Assistance. Proceedings of the IEEE Conference on Advancements in Computational Sciences.

[55] Alonazi M., Ansar H., Al Mudawi N., Alotaibi S.S., Almujally N.A., Alazeb A. (2023). Smart Healthcare Hand Gesture Recognition Using CNN-Based Detector and Deep Belief Network. IEEE Access.

[56] Ansar H., Ksibi A., Jalal A., Shorfuzzaman M., Alsufyani A., Alsuhibany S.A., Park J. (2022). Dynamic Hand Gesture Recognition for Smart Lifecare Routines via K-Ary Tree Hashing Classifier. Appl. Sci..

[57] Faisal A., Jalal A. Multi-Pedestrians Anomaly Detection via Conditional Random Field and Deep Learning. Proceedings of the 2023 4th International Conference on Advancements in Computational Sciences (ICACS).

[58] Almujally N.A., Khan D., Al Mudawi N., Alonazi M., Alazeb A., Algarni A., Jalal A., Liu H. (2024). Biosensor-Driven IoT Wearables for Accurate Body Motion Tracking and Localization. Sensors.

[59] Mudawi N.A., Pervaiz M., Alabduallah B.I., Alazeb A., Alshahrani A., Alotaibi S.S., Jalal A. (2023). Predictive Analytics for Sustainable E-Learning: Tracking Student Behaviors. Sustainability.

[60] Al Mudawi N., Tayyab M., Ahmed M.W., Jalal A. Machine Learning Based on Body Points Estimation for Sports Event Recognition. Proceedings of the 2024 IEEE International Conference on Autonomous Robot Systems and Competitions (ICARSC).

[61] Ahmad J., Amir N., Bobasu S. Human Body Parts Estimation and Detection for Physical Sports Movements. Proceedings of the 2019 2nd International Conference on Communication, Computing and Digital systems (C-CODE).

[62] Nida K., Gochoo M., Ahmad J., Kim K. (2021). Modeling Two-Person Segmentation and Locomotion for Stereoscopic Action Identification: A Sustainable Video Surveillance System. Sustainability.

[63] Alabdullah B., Tayyab M., AlQahtani Y., Al Mudawi N., Algarni A., Jalal A., Park J. (2023). Sports Events Recognition Using Multi Features and Deep Belief Network. Comput. Mater. Contin..

[64] Zhang S., Wang C., Zhang H., Lin H. (2024). Collective Dynamics of Adaptive Memristor Synapse-Cascaded Neural Networks Based on Energy Flow. Chaos Solitons Fractals.

[65] Wang K., Boonpratatong A., Chen W., Ren L., Wei G., Qian Z., Lu X., Zhao D. (2023). The Fundamental Property of Human Leg During Walking: Linearity and Nonlinearity. IEEE Trans. Neural Syst. Rehabil. Eng..

[66] Xing J., Yuan H., Hamzaoui R., Liu H., Hou J. (2023). GQE-Net: A Graph-Based Quality Enhancement Network for Point Cloud Color Attribute. IEEE Trans. Image Process..

[67] Ding J., Chen X., Lu P., Yang Z., Li X., Du Y. (2023). DialogueINAB: An Interaction Neural Network Based on Attitudes and Behaviors of Interlocutors for Dialogue Emotion Recognition. J. Supercomput..

[68] Zheng W., Lu S., Yang Y., Yin Z., Yin L. (2024). Lightweight Transformer Image Feature Extraction Network. PeerJ Comput. Sci..

[69] Wang D., Yang S.X. (2023). Broad Learning System with Takagi–Sugeno Fuzzy Subsystem for Tobacco Origin Identification Based on Near-Infrared Spectroscopy. Appl. Soft Comput..

[70] Yin L., Wang L., Lu S., Wang R., Yang Y., Yang B., Liu S., AlSanad A., AlQahtani S.A., Yin Z. (2024). Convolution-Transformer for Image Feature Extraction. Comput. Model. Eng. Sci..

[71] Hou X., Xin L., Fu Y., Na Z., Gao G., Liu Y., Xu Q., Zhao P., Yan G., Su Y. (2023). A Self-Powered Biomimetic Mouse Whisker Sensor (BMWS) Aiming at Terrestrial and Space Objects Perception. Nano Energy.

[72] Ding Y., Zhang W., Zhou X., Liao Q., Luo Q., Ni L.M. (2021). FraudTrip: Taxi Fraudulent Trip Detection from Corresponding Trajectories. IEEE Internet Things J..

[73] Shen X., Jiang H., Liu D., Yang K., Deng F. (2022). PupilRec: Leveraging Pupil Morphology for Recommending on Smartphones. IEEE Internet Things J..

[74] Wang E., Yang Y., Wu J., Liu W., Wang X. (2018). An Efficient Prediction-Based User Recruitment for Mobile Crowdsensing. IEEE Trans. Mob. Comput..

[75] Wang X., Zhang R., Miao Y., An M., Wang S., Zhang Y. (2024). PI^2^-Based Adaptive Impedance Control for Gait Adaption of Lower Limb Exoskeleton. IEEE/ASME Trans. Mechatron..

[76] Zhou Y., Xie J., Zhang X., Wu W., Kwong S. (2024). Energy-Efficient and Interpretable Multisensor Human Activity Recognition via Deep Fused Lasso Net. IEEE Trans. Emerg. Top. Comput. Intell..

[77] Mi C., Liu Y., Zhang Y., Wang J., Feng Y., Zhang Z. (2023). A Vision-Based Displacement Measurement System for Foundation Pit. IEEE Trans. Instrum. Meas..

[78] He S., Chen W., Wang K., Luo H., Wang F., Jiang W., Ding H. (2024). Region Generation and Assessment Network for Occluded Person Re-Identification. IEEE Trans. Inf. Forensics Secur..

[79] Zhou Z., Wang Y., Liu R., Wei C., Du H., Yin C. (2022). Short-Term Lateral Behavior Reasoning for Target Vehicles Considering Driver Preview Characteristic. IEEE Trans. Intell. Transp. Syst..

[80] Liu Q., Yuan H., Hamzaoui R., Su H., Hou J., Yang H. (2021). Reduced Reference Perceptual Quality Model With Application to Rate Control for Video-Based Point Cloud Compression. IEEE Trans. Image Process..

[81] Srivastava N., Mansimov E., Salakhudinov R. Unsupervised Learning of Video Representations Using LSTMs. Proceedings of the 32nd International Conference on Machine Learning.

[82] Akhter I., Jalal A., Kim K. Pose Estimation and Detection for Event Recognition Using Sense-Aware Features and AdaBoost Classifier. Proceedings of the 2021 International Bhurban Conference on Applied Sciences and Technologies (IBCAST).

[83] Meng Q., Zhu H., Zhang W., Piao X., Zhang A. (2020). Action Recognition Using Form and Motion Modalities. ACM Trans. Multimed. Comput. Commun. Appl..

[84] De Siva N.H.T.M., Rupasingha R.A.H.M. Classifying YouTube Videos Based on Their Quality: A Comparative Study of Seven Machine Learning Algorithms. Proceedings of the 2023 IEEE 17th International Conference on Industrial and Information Systems (ICIIS).

[85] de Almeida Maia H., Concha D.T., Pedrini H., Tacon H., de Souza Brito A., de Lima Chaves H., Vieira M.B., Villela S.M. (2020). Action Recognition in Videos Using Multi-stream Convolutional Neural Networks. Deep Learning Applications.

[86] Akhter I., Jalal A., Kim K. (2020). Adaptive Pose Estimation for Gait Event Detection Using Context-aware Model and Hierarchical Optimization. J. Electr. Eng. Technol..

[87] Abdulazeem Y., Balaha H.M., Bahgat W.M., Badawy M. (2021). Human Action Recognition Based on Transfer Learning Approach. IEEE Access.

[88] Shrestha M., Pandey S.P. Human Action Recognition using Deep Learning Methods. Proceedings of the 14th IOE Graduate Conference.

